# Modern Sociology

**Published:** 1903-06-06

**Authors:** 


### Modern Sociology.

### A MUNICIPAL MUSEUM.

When, on the passing of the London Government Act of 1899, the powers of the Commissioners for public libraries and museums in Whitechapel were transferred to the Borough Council of Stepney, one of the most interesting of their acquirements was the museum in Whitechapel Road. The nucleus of the museum was a collection made by the Rev. Dan Greatorex, late vicar of St. Paul's Church, bequeathed to Whitechapel, and housed under the roof of the Public Library. Since its foundation, over half a million persons have visited it, and something like sixty free popular lectures have been given by men of eminence in the scientific world. Many of the visitors are men who come in during the dinner hour, or after they leave work, or on public holidays (the museum is open on Sundays and Bank Holidays from three to ten), a few are women, and a great many are children. And here comes in its special interest from an educational point of view, for the collection is used to demonstrate elementary natural history, physiology, and other scientific subjects to the children attending the public schools in the Tower Hamlets division. Many of the teachers value it very highly, and take pains to prepare the children before the periodical visits, thus greatly enhancing the value of the demonstration.

These visits count as school time, and the children come to the museum under the charge of their teachers. On the day of a special visit made with a view to seeing how the work was carried out, there were 30 children present from a neighbouring school, all girls of Standard IV. They were divided into three groups. The lesson was on the circulation of the blood, and one of the teachers was provided with a large model of a heart, and the other with coloured diagrams, while the curator of the museum, Miss Kate Hall, demonstrated with a sheep's heart. The movement of blood in the web of a frog's foot completes this lesson. By changing the groups each child was shown the diagrams, the model, and the real object. They appeared greatly interested, and the teachers evidently enjoyed the opportunity of using good models. The whole lesson lasted three-quarters of an hour.

Botany and natural history lessons are given in the same way, and for these live ants, bees, and frogs are kept, as well as fresh flowers and preserved specimens. As a proof that such instruction is needed, one small boy, being asked what he supposed the tiny red objects running about in the ant-case might be, replied, without hesitation, "Fleas!" The ant-case is arranged after the plan approved by Lord Avebury, and there is also a large ant-hill. Sometimes it is possible for the curator to visit some of the schools and give a demonstration there, and the arrangement of temporary exhibitions at other libraries in the Borough forms part of her work. These are no doubt useful in making the work of the museum known, but concentration on a good central collection is probably a wiser aim, and likely to result in more permanent usefulness.

The monthly exhibition of "Nature notes," as they might be called, is one of the most educational ideas in this museum. Migration, for example, was the subject recently shown. One or two specimens of typical migrants were chosen from the collection, and some clearly printed notes on migration accompanied them, the whole being raised in a centre case, not too high for children to look at comfortably. The natural history and geography of the plum pudding was another winter subject. A space is kept for the flowers of the month, and during the season the London School Board kindly contributes a weekly box of flowers and other botanical specimens. Contributions of flowers would be greatly valued.

In the arrangement of the specimens, the aim is to keep broad and simple ideas before the visitors. Just inside the door, one comes upon a wall case containing specimens of the palæozoic, mesozoic, and tertiary periods, leading up to man and some of the things he makes in his various stages of development—*e.g.* primitive weapons, a Samoan Kava bowl, Egyptian gods. A series of natural history cases comes next, and occupies three sides of the room, until, passing cases of invertebrates and vertebrates, including some beautifully stuffed birds, man is again reached in the shape of a human skeleton.

The fishes have proved of great interest to the various fishing clubs that abound in East and North London. The mounting of the butterflies, which helped to gain a bronze medal at the Nature Study Exhibition last July, is particularly artistic. They are put in natural positions on the twigs of trees they mostly affect, and the various stages are shown —egg, caterpillar and chrysalis, each group being distinct. The same idea of simplicity and of keeping as close to nature as possible is obvious in the bee case, where a clover-flower, not pressed, but successfully dried, has a humble bee on it, and a note states that the humble bee is the only one whose tongue is long enough to suck the red clover honey. A beautiful lesson on Wings is in course of preparation, and Miss Hall's model of the structure of a feather is exquisitely neat. The mounting of the wings of a wild duck, with every feather in place, is a work of art and great patience.

Some valuable relics of the ancient Liberty of Norton Folgate have been secured, and that a municipal museum should be, primarily, a safe repository for all objects of local antiquity and permanent historic interest, is the view of the curator. After the local collection, she says, the authorities may choose to instruct and interest the people in natural history, arts, or crafts, and the aim of the collection should be "to attract judiciously the ordinary visitor, and inspire him with an interest and enthusiasm for the subject illustrated, and not weary him with details. These, however, should be at hand for students and for demonstration purposes, in the form of a reserve collection kept in drawers." Butterflies, eggs, and a herbarium are treated as reserve collections at Stepney

In a report on the work and aims of the museum, the curator remarks that a local museum in London should aim only at being a stepping-stone to the better understanding of our larger national museums. In the arrangement of the collections, the needs of the locality should be most carefully considered. It is possible that natural history would form one of the most valuable means of general instruction and education, and especially so in East London, "where man has crowded out most other living things." This is a branch that the committee is desirous of expanding, and the adaptation of an available space for a Nature Study centre is under consideration. Great things, however, cannot be looked for on a library rate; by adopting the Museums and Gymnasiums Act (1891), however, a separate rate could be levied, and whereas a rate of one-tenth of a penny in the pound would only be felt to the extent of fivepence yearly by the £50 householder, it would bring in something like £500 a year to the museum.

Judging by what is being done in Stepney, the subject of municipal museums is one that merits the attention of all London Borough Councils, especially if the rumour that they are to be the educational authorities of the future should prove true.

